# The value of using the faecal immunochemical test in general practice on patients presenting with non-alarm symptoms of colorectal cancer

**DOI:** 10.1038/s41416-018-0178-7

**Published:** 2018-08-01

**Authors:** Jakob Søgaard Juul, Nete Hornung, Berit Andersen, Søren Laurberg, Frede Olesen, Peter Vedsted

**Affiliations:** 1 Department of Public Health, Research Unit for General Practice, Aarhus University, Bartholins Allé 2, 8000 Aarhus C, Denmark; 2 Department of Public Health, Research Centre for Cancer Diagnosis in Primary Care, Aarhus University, Bartholins Allé 2, 8000 Aarhus C, Denmark; 3 Department of Clinical Biochemistry, Randers Regional Hospital, Skovlyvej 1, 8930 Randers, NE Denmark; 4 Department of Public Health Programmes, Randers Regional Hospital, Skovlyvej 1, 8930 Randers, NE Denmark; 5 Department of Surgery, Aarhus University Hospital, Tage Hansens Gade 2, 8000 Aarhus C, Denmark; 6 Department of Clinical Medicine, Diagnostic Centre, University Research Clinic for Innovative Patient Pathways, Silkeborg Regional Hospital, Aarhus University, Aarhus C, Denmark

**Keywords:** Digestive signs and symptoms, Colorectal cancer, Diagnosis

## Abstract

**Background:**

Around 50% of individuals with colorectal cancer (CRC) initially present with non-alarm symptoms.

**Methods:**

We investigated the value of using the faecal immunochemical test (FIT) in the diagnostic process of CRC and other serious bowel disease in individuals presenting with non-alarm symptoms in general practice. The study was conducted in the Central Denmark Region from 1 September 2015 to 30 August 2016. The FIT was used as a rule-in test on patients aged ≥30 years with non-alarm symptoms of CRC. The cut-off value was set to 10 µg Hb/g faeces.

**Results:**

A total of 3462 valid FITs were performed. Of these, 540 (15.6%) were positive. Three months after FIT performance, 51 (PPV: 9.4% (95% CI: 7.0;11.9)) individuals with a positive FIT were diagnosed with CRC and 73 (PPV: 13.5% (95%CI: 10.6;16.4)) with other serious bowel disease. Of CRCs, 66.7% were diagnosed in UICC stage I & II and 19.6% in stage IV. The false negative rate for CRC was <0.1% for the initial 3 months after FIT performance.

**Conclusion:**

The FIT may be used as a supplementary diagnostic test in the diagnostic process of CRC and other serious bowel disease in individuals with non-alarm symptoms of CRC in general practice.

## Introduction

Colorectal cancer (CRC) is the third most common cancer worldwide and a major reason for cancer-related death.^1^ However, CRC is potentially curable if found in early stages.^2^ Screening for CRC and urgent referral in a Cancer Patient Pathway (CPP) for patients presenting alarm symptoms of CRC are two important strategies used to support early diagnosis of CRC.^3–7^ However, despite screening, the majority of new CRC cases must be found on symptomatic presentation in general practice, and ∼50% of these patients will present symptoms and signs that do not qualify for urgent referral.^8–10^ These low-risk symptoms or “non-alarm symptoms” are a heterogeneous group of uncharacteristic and vague symptoms that most often are signs of benign conditions.^11^ For these patients, the GP will often use a “wait and see” and safety netting approach, which is reflected in a longer diagnostic process compared to patients with alarm symptoms. This may lead to stage progression and ultimately to poorer prognosis.^12–18^ In addition, individuals with CRC has been shown to consult their GP more in the year preceding diagnosis compared with matched patients.^19^ Thus, new diagnostic strategies could contribute to aid the GP in the diagnostic workup of patients with non-alarm symptoms of CRC.

One option may be the faecal immunochemical test (FIT). The test detects microscopic blood in faeces and is shown to have better sensitivity for detecting CRC than the guaiac faecal occult blood test (gFOBT) and alarm symptoms.^20–22^ A range of studies have indicated that the FIT may benefit the triage of patients at risk of CRC.^22–30^ In the UK, an updated version of the National Institute for Health and Care Excellence (NICE) guidelines have suggested faecal occult testing on individuals with low-risk symptoms.^31^ This was followed by the DG30 guidance that provided an evidence-based guide for the use of FIT in general practice.^32^ However, no previous study has examined whether the FIT would actually be of value in the diagnostic workup of these individuals.‬‬‬‬‬‬‬‬

Therefore, we aimed to investigate in a large-scale study the value of using the FIT in general practice on patients presenting with non-alarm symptoms of CRC.

## Materials and methods

### Design

The study was designed as a prospective cohort study and based on the establishment of access to the FIT for GPs in the Central Denmark Region.^33^ The study took place from 1 September 2015 to 30 August 2016.

### Setting and study participants

The Central Denmark Region has ∼853 GPs working in 385 general practices. GPs in Denmark own their own practice, and 99% of Danish citizens are registered with a general practice.^34^ A GP has ∼1550 persons listed and acts as gatekeeper to secondary care. Before this study, Danish GPs did not have systematic access to the FIT from general practice. Thus, GPs were provided with the possibility of requesting FIT from their clinic, and a logistic setup was arranged to enable analysis of the FITs from general practice. Furthermore, a training course on FIT use was arranged to teach the GPs about the aim of using the FIT and the precise target group for the faecal immunochemical testing.^35^

We included all individuals aged ≥30 years who had performed a valid FIT (defined as a FIT result within the measuring range of the OC Sensor DIANA) in general practice during the study period. Included individuals were followed up from the day of FIT request until 3 months after. A follow-up time of 3 months was used because individuals with a positive FIT should be urgently referred to diagnostic investigation. Invalid FIT results were defined as a FIT without a quantified value and excluded from analyses. Only one FIT per individual was included. This was defined as; either the latest performed FIT or the FIT requested immediately before the referral to diagnostic investigation (sigmoidoscopy, colonoscopy or computed tomography (CT) colonography) as this FIT was assumed to be decisive for further investigation.

### Use of the faecal immunochemical test in general practice

According to the Danish CPP for CRC, individuals aged ≥40 years should be urgently referred to colonoscopy if they present with alarm symptoms. These include: rectal bleeding, change in bowel habits >4 weeks, abdominal pain and iron deficiency anaemia. However, the literature shows that symptoms and signs of disease can take different form of severity (“the symptom continuum”) and that interpretation of alarm symptoms vary between GPs.^36–38^ Therefore, faecal immunochemical testing was aimed at individuals aged ≥30 years who presented in general practice with non-alarm symptoms of CRC. It was left to the GPs’ clinical knowledge and judgement to decide on which patients to request a FIT, but GPs were provided with a clinical instruction containing suggested symptoms and signs. These included: change in bowel habits, abdominal pain, unexplained anaemia, and unspecific symptoms (e.g. fatigue or weight loss). Furthermore, faecal immunochemical testing was recommended as part of the diagnostic work up of irritable bowel syndrome (IBS). It was a strict prerequisite for using the FIT that the GP did not interpret the patient’s symptoms as eligible for urgent referral in the CPP for CRC as these patients should not be delayed by performance of a FIT.^38^ The rationale behind which symptoms and signs to include in the clinical instruction has been presented previously in a separate article.^33^

The GPs requested the FITs through the usual online ordering system for laboratory tests, WebReq, and registered the indications for requesting the FIT by ticking a box on a list of symptoms and signs from the clinical instruction. GPs could also tick a box labelled “other” if the FIT was requested on symptoms or signs other than the ones stated in the instruction. The FIT was used as a rule-in test, and the cut-off value for a positive FIT in general practice was set at 10 μg Hb/g faeces. Thus, a positive FIT should imply urgent referral to colonoscopy, whereas a negative test could guide the GP in the direction of the most appropriate diagnostic strategy alongside with continued safety netting.

A single FIT sample was collected from each patient containing 10 mg faeces in 2 ml buffer solution. The FITs were sent with prioritised mail for analyses to the Department of Clinical Biochemistry at Randers Regional Hospital. The FITs were analysed daily by trained staff with expertise in FIT analyses, using the automated analyser OC-Sensor DIANA (Eiken Chemical Company, Ltd, Japan). FIT results were stored on the department’s server and returned electronically to the GPs. The FIT used was a quantitative test and the coefficient of variation (CV%) of the assay was <5%, and the measuring range was 7–200 µg Hb/g faeces (stated as <7 μg Hb/g faeces for faecal haemoglobin concentrations below the detection limit). The staff performing the analysis of the FIT at the Department of Clinical Biochemistry at Randers Regional Hospital were blinded to the project. The doctors performing the colonoscopy were not blinded to FIT results, but had no affiliation with the project.

### Outcome measures

1. *Number of requested FITs*.
2. *FIT results*. Defined as: positive (≥10 μg Hb/g faeces), negative (≤9μg Hb/g faeces) or invalid.
3. *Diagnostic investigations after the FIT request*. Defined as: sigmoidoscopy, colonoscopy or CT-colonography.
4. *Diagnoses after the FIT request*. This was the primary outcome of the study. Diagnoses of interest were: CRC or other serious bowel disease (SBD). SBD was defined as: diagnosis of either inflammatory bowel disease (IBD) or high-risk adenomas (HRA). According to the literature, high-risk adenomas were defined as: high-grade dysplasia, size ≥ 1 cm or ≥3 adenomas.^39,40^
5. *Stage and location of CRC*. Stages of CRC were defined by the international standard for staging CRC, i.e. Union for International Cancer Control (UICC) staging.^41^ The location of CRC was categorised into: proximal colon (caecum, ascending colon or transverse colon), distal colon (descending colon and sigmoid colon), or rectum.
6. *Symptoms and signs reported for requesting FITs*. Distribution, rate of positive FITs, and the positive predictive values (PPVs) for CRC and SBD for symptoms and signs registered by the GPs.
7. *Rate of positive FITs and PPVs for CRC or SBD at different age and gender*. The PPV was estimated for ordering the FIT and for a positive FIT (≥10 μg Hb/g faeces).
8. *PPVs for detecting CRC and SBD at different faecal haemoglobin concentrations*. These were categorised into four intervals: 10–19 μg Hb/g faeces, 20–99 μg Hb/g faeces, 100–199 μg Hb/g faeces, and ≥200 μg Hb/g faeces.

### Sample size

We expected ∼33,600 FITs to be requested during the study period, corresponding to 1–2 FITs requested per week per GP in the region. The positivity rate was assumed to be ∼10%, which is slightly higher than in the Danish screening programme.^42^ After assessing the literature on performance of the FIT in both symptomatic patients and in screening, we expected an overall PPV for CRC of ∼10% when the FIT was positive. Thus, in total, we expected 336 CRCs to be diagnosed during the study period.

### Data collection

The Danish civil registration number was used to link registers used in the study.^43^ The FIT results were delivered electronically by the Department of Clinical Biochemistry at Randers Regional Hospital, together with the indications for using the FIT.

Data on socioeconomic position were collected from Statistics Denmark and the level of comorbidity was obtained by the Charlson Comorbidity Index (CCI).^44,45^ Data on diagnostic investigations were gathered from the Danish National Patient Register and the Danish National Health Service Register.^46,47^ Diagnoses on CRC, IBD, and HRA were obtained from the Danish Pathology Register.^48^ Data on CRC stages were collected from the Danish National Patient Register, and this was supplemented by information from the electronic patient records.

### Statistical analysis

The PPVs for CRC and SBD were assessed for all individuals aged ≥30 years who had performed valid FIT during the study period. To avoid overestimation, the PPVs for CRC and SBD after a positive FIT were calculated using all individuals with a positive FIT in the denominator. Likewise, the false negative rate was calculated using all individuals with a negative test in the denominator. Analyses of PPVs for CRC and SBD were stratified for gender and age as these two factors have been shown to act as effect modifiers.^49^ Furthermore, PPVs were also investigated for different faecal haemoglobin concentrations to assess if there was a lower limit of blood in faeces for which diagnosis was unlikely. For these analyses, we stratified the faecal haemoglobin concentrations into four intervals: 10–19, 20–99, 100–199 and ≥200 μg Hb/g faeces. *P*-values were calculated by Fisher’s exact test.

To meet the international recommendation, the faecal haemoglobin concentrations were reported in µg Hb/g faeces.^50^ According to the manufacturer, the OC Sensor DIANA collects an average of 10 mg faeces and contains 2 ml buffer.

All analyses were performed on the server of Statistics Denmark using Stata 14. Due to the regulations on anonymous data reporting, we could not report data containing less than three observations.

### Approvals

The study obtained ethical clearance from the Committee on Health Research Ethics in the Central Denmark Region (j. no. 142/2014) and was approved by the Danish Data Protection Agency (j. no. 2015-41-3913). The Danish Health and Medicines Authority gave legal permission to obtain information from patient records (3-3013-1026-1). The study was registered at clinicaltrials.gov (NCT02308384, date of registration: 26 November 2014).

## Results

**Fig. 1:**
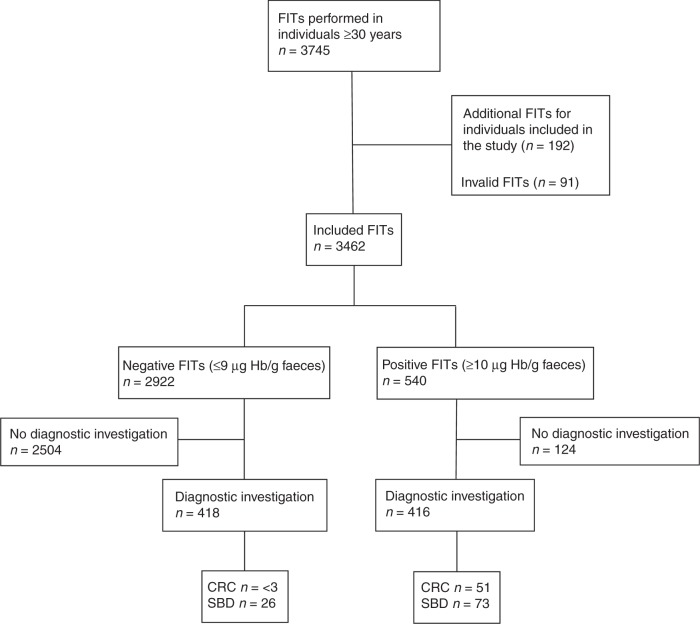
Flow chart of FIT requisitions in the study period

During the study period, 3745 FITs were requested. Of these, 91 (2.4%) FITs were invalid and 192 (5.1%) additional FITs were excluded to ensure only one test per individual. Thus, a total of 3462 (92.5%) FITs were included in the analyses. Of the included FITs, 2921 (84.4%) were negative and 540 (15.6%) were positive (Fig. 1). The characteristics of tested individuals are shown in Table 1. Three months after requisition, diagnostic investigation had been performed in 416 (77.0%) of individuals with a positive FIT and 418 (14.3%) with a negative FIT (Table 2). Among all individuals with a positive FIT, 51 (9.4%) were diagnosed with CRC and 73 (13.5%) with SBD (11 with IBD and 62 with HRA). Less than three (<0.1%) CRCs and 26 (0.9%) cases of SBD (20 IBDs and 6 HRAs) were found among individuals with a negative test. No individuals without a registered diagnostic investigation had a diagnosis of either CRC or SBD within 3 months after performance of the FIT, suggesting no emergency presentations during the study period.

**Table 1:** Characteristics of individuals included in the study

	*n* = 3462 (%)
*Age (years)*	
30–39	228 (6.6)
40–49	620 (17.9)
50–59	723 (20.9)
60–69	877 (25.4)
70–79	701 (20.2)
≥80	313 (9.0)
*Gender*	
Female	1942 (56.1)
Male	1520 (43.9)
*Country of origin*	
Danish	3280 (94.8)
Immigrant—western country	84 (2.4)
Immigrant—non-western country	98 (2.8)
*Educational level*	
Basic	1024 (29.6)
Medium	1594 (46.0)
High	844 (24.4)
*Labour market affiliation* ^a^	
Working	1649 (47.8)
Unemployed	185 (5.4)
Retirement pension	1618 (46.8)
*Marital status* ^b^	
Married/cohabiting	2428 (70.5)
Living alone	1018 (29.5)
*Charlson Comorbidity Index*	
Low (CCI score = 0)	2443 (70.5)
Moderate (CCI score = 1–2)	768 (22.2)
Severe (CCI score ≥ 3)	251 (7.3)

^a^Information on labour market information was missing for 10 individuals.

^b^Individuals Information on marital status was missing for 16 individuals

**Table 2:** Diagnostic investigations and diagnoses 3 months after performance of FIT

	Positive FITs (≥10 μg Hb/g faeces)		Negative FITs (<9 μg Hb/g faeces)	
	*n*	% (95%CI)	*n*	% (95%CI)
Requested FITs	540	15.6 (14.4;16.8)	2922	84.4 (83.2;85.6)
*Diagnostic investigation*				
Colonoscopy/CT colonography	416	77.0	418	14.3
No diagnostic investigation	124	23.0	2504	85.7
*Diagnoses*				
Colorectal cancer or serious bowel disease	124	23.0 (19.4;26.5)	NA	–
Serious bowel disease	73	13.5 (10.6;16.4)	26	0.9 (0.5;1.2)
Inflammatory bowel disease	11	2.0 (0.8;3.2)	20	0.7 (0.4;1.0)
High risk adenoma	62	11.5 (8.8;14.2)	6	0.2 (0.1;0.4)
Colorectal cancer	51	9.4 (7.0;11.9)	<3	<0.1
Location				
Proximal colon	21	41.2 (27.2;55.2)	NA	–
Distal colon	16	31.4 (18.2;44.6)	NA	–
Rectum	14	27.5 (14.8;40.1)	NA	–
* UICC stage*				
Stage I	13	25.5 (13.1;37.9)	NA	–
Stage II	21	41.2 (27.2;55.2)	NA	–
Stage III	7	13.7 (4.0;23.5)	NA	–
Stage IV	10	19.6 (8.3;30.9)	NA	–

Of the 51 CRCs diagnosed after a positive FIT, 34 (66.7%) were detected in UICC stage I and II, and 10 (19.6%) in stage IV. More CRCs were located in the proximal colon (41.2%) than in the distal colon (31.4%) or the rectum (27.4%).

### Indications for using the FIT

The distribution of indications for requesting the FIT are shown in Table 3. In total, 1169 (33.7%) individuals had one indication reported, whereas 780 (22.5%) individuals had three or more. No indication was reported in 348 (10.2%) individuals. The most frequently reported symptoms or signs were change in bowel habits (53.9%) and abdominal pain (45.6%).

**Table 3:** Symptoms and signs reported by the GPs when requesting the FIT

	All FITs		Positive FITs		CRC after positive FIT		SBD after positive FIT	
	(*n* = 3462)		(*n* = 540)		(*n* = 51)		(*n* = 73)	
	*n*	%	*n*	% (95%CI)	*n*	PPV (95%CI)	*n*	PPV (95%CI)
*Individual symptom and signs*								
Abdominal pain	1579	45.6	210	13.3 (11.6;15.0)	18	8.6 (4.8;12.4)	17	8.1 (4.4;11.8)
Change in bowel habits	1867	53.9	290	15.5 (13.9;17.2)	27	9.3 (5.9;12.7)	34	11.7 (8.0;15.4)
Uncharacteristic symptoms	827	23.9	139	16.8 (14.3;19.4)	11	7.9 (3.4;12.5)	14	10.1 (5.0;15.1)
Unexplained Anaemia	424	12.3	87	20.5 (16.7;24.4)	10	11.5 (4.7;18.3)	8	9.2 (3.0;15.4)
Investigation for IBS	776	22.4	103	13.3 (10.9;15.7)	8	7.8 (2.5;13.0)	12	11.7 (5.3;18.0)
Other	586	16.9	89	15.2 (12.3;18.1)	5	5.6 (0.7;10.5)	19	21.3 (12.7;30.0)
No indication reported	348	10.2	63	–	7	–	15	–
*Multiple symptoms and signs*								
1 symptom	1169	33.7	176	15.1 (13.0;17.1)	20	11.4 (6.6;17.0)	24	13.6 (8.5;18.8)
2 symptoms	1165	33.6	191	16.4 (14.3;18.5)	15	7.9 (4.0;11.7)	23	12.0 (7.4;16.7)
≥3 symptoms	780	22.5	110	14.1 (11.7;16.6)	9	8.2 (3.0;13.4)	11	10.0 (4.3;15.7)
No indication reported	348	10.2	63	–	7	–	15	–

The GP could register more than one indication for each patient

Interestingly, 20.5% (95% confidence interval (95%CI): 16.7;24.4) of the individuals with unexplained anaemia had a positive FIT. For the remaining symptoms and signs, the rate of positive FITs was in the range 13–17%.

The PPV for CRC was highest for unexplained anaemia (11.5% (95%CI: 4.7;18.3)) and change in bowel habits (9.3% (95%CI: 5.9;12.7)). For SBD, the highest PPV was found for “other” symptoms (21.3% (95%CI: 12.7;30.0)). The PPV for CRC and SBD when having a positive test and one indication was 11.4% (95%CI: 6.6;17.0) and 13.6% (95%CI: 8.5;18.8), respectively. For three or more indications, the corresponding figures were 8.2% (95%CI: 3.0;13.4) and 10.0% (95%CI: 4.3;15.7), respectively.

### Rate of positive FITs, and PPV for detecting CRC and SBD for different age and gender

More females (1942 (56.1%)) than males (1520 (43.9%)) had a FIT performed (Table 4).

**Table 4:** Numbers of FITs requested, positive FITs (cut-off 10 μg Hb/g faeces), and diagnosed CRCs and other serious bowel disease (SBD) after a positive FIT, stratified for gender and age groups. Positive predictive values (PPV) are given for CRC and SBD when the GP decided to request FIT and when FIT was positive

	Requested FITs	Positive FITs	CRCs after a positive FIT	SBD after a positive FIT	Rate of positive FITs	PPV for CRC when the GP requested the FIT	PPV for SBD when the GP requested the FIT	PPV for CRC if the FIT was positive	PPV for SBD if the FIT was positive
	*n*	*n*	*n*	*n*	% (95%CI)	PPV (95%CI)	PPV (95%CI)	PPV (95%CI)	PPV (95%CI)
*30–39 years*									
All	228	30	0	NA	13.2 (8.7;17.6)	0	NA	0	NA
Males	101	9	0	0	8.9 (3.3;14.6)	0	0	0	0
Females	127	21	0	NA	16.5 (10.0;23.1)	0	NA	0	NA
*40–49 years*									
All	620	57	4	NA	9.2 (6.9;11.5)	0.6 (0.1;1.3)	NA	7.0 (1.8;13.9)	NA
Males	269	19	NA	NA	7.1 (4.0;10.1)	NA	NA	NA	NA
Females	351	38	NA	NA	10.8 (7.6;14.1)	NA	NA	NA	NA
*50–59 years*									
All	723	79	5	9	10.9 (8.6;13.2)	0.7 (0.1;1.3)	1.2 (0.4;2.1)	6.3 (0.8;11.8)	11.4 (4.2;18.6)
Males	323	43	NA	4	13.3 (9.6;17.0)	NA	1.2 (0.1;2.5)	NA	9.3 (0.2;18.3)
Females	400	36	NA	5	9.0 (6.2;11.8)	NA	1.3 (0.2;2.3)	NA	13.9 (2.0;25.8)
*60–69 years*									
All	877	129	14	17	14.7 (12.4;17.1)	1.6 (0.8;2.4)	1.9 (1.0;2.9)	10.9 (5.4;16.3)	13.2 (7.3;19.1)
Males	382	69	10	9	18.1 (14.2;21.9)	2.6 (1.0;4.2)	2.4 (0.8;3.9)	14.5 (6.0;23.0)	13.0 (4.9;21.2)
Females	495	60	4	8	12.1 (9.3;15.0)	0.8 (0.1;1.6)	1.6 (0.5;2.7)	6.7 (0.2;13.2)	13.3 (4.5;22.2)
*70–79 years*									
All	701	155	15	28	22.1 (19.0;25.4)	2.1 (1.1;3.2)	4.0 (2.5;5.4)	9.7 (5.0;14.4)	18.1 (11.9;24.2)
Males	304	70	9	11	23.0 (18.3;27.8)	3.0 (1.0;4.9)	3.6 (1.5;5.7)	12.9 (4.8;20.9)	15.7 (7.0;24.5)
Females	397	85	6	17	21.4 (17.4;25.5)	1.5 (0.3;2.7)	4.3 (2.3;6.3)	7.1 (1.5;12.6)	20.0 (11.3;28.7)
*≥80 years*									
All	313	90	13	13	28.8 (23.7;33.8)	4.2 (1.9;6.4)	4.2 (1.9;6.4)	14.4 (7.0;21.8)	14.4 (7.0;21.8)
Males	141	45	9	6	31.9 (24.1;39.7)	6.4 (2.3;10.5)	4.3 (0.9;7.6)	20.0 (7.8;32.2)	13.3 (3.0;23.7)
Females	172	45	4	7	26.2 (19.5;32.8)	2.3 (0.1;4.6)	4.1 (1.1;7.1)	8.9 (0.2;17.5)	15.6 (4.5;26.6)
*Total*									
All	3462	540	51	73	15.6 (14.4;16.8)	1.5 (1.1;1.9)	2.1 (1.6;2.6)	9.4 (7.0;11.9)	13.5 (10.6;16.4)
Males	1520	255	34	31	16.8 (14.9;18.7)	2.2 (1.5;3.0)	2.0 (1.3;2.8)	13.3 (9.1;17.5)	12.2 (8.1;16.2)
Females	1942	285	17	42	14.7 (13.1;16.2)	0.9 (0.5;1.3)	2.2 (1.5;2.8)	6.0 (3.2;8.7)	14.7 (10.6;18.9)

The overall rate of positive FITs was slightly higher for males (16.8% (95%CI: 14.9;18.7)) than for females (14.7% (95%CI: 13.1;16.2)). For males, the rate of positive FITs increased with age, whereas a U-shaped trend was observed among females with a high rate of positive tests among the 30–39 year old (16.5% (95%CI: 10.0;23.1)) (Table 4).

The overall PPV for CRC when the GP decided to request a FIT was 1.5% (95%CI: 1.1;1.9) and 9.4% (95%CI: 7.0;11.9) if the FIT was positive (Table 4). For SBD the PPV was 2.1% (95%CI: 1.6;2.6) when requesting the FIT and 13.5% (95%CI: 10.6;16.4) when the FIT was positive. In general, the PPV for detecting either CRC or SBD increased with age, but no CRCs were found in individuals aged <40 years. Interestingly, females had a significantly higher PPV for SBD than CRC (SBD: 14.7% (95%CI: 10.6;18.9) vs. CRC: 6.0% (95%CI: 3.2;8.7) (*p* < 0.01)), whereas males were more often diagnosed with CRC than SBD (CRC: 13.3% (95%CI: 9.1;17.5) vs. SBD: 12.2% (95%CI: 8.1;16.2)) and had significantly higher PPV for CRC than females (*p* < 0.01)).

### PPVs for detecting CRC and SBD at different faecal haemoglobin concentrations

**Fig. 2:**
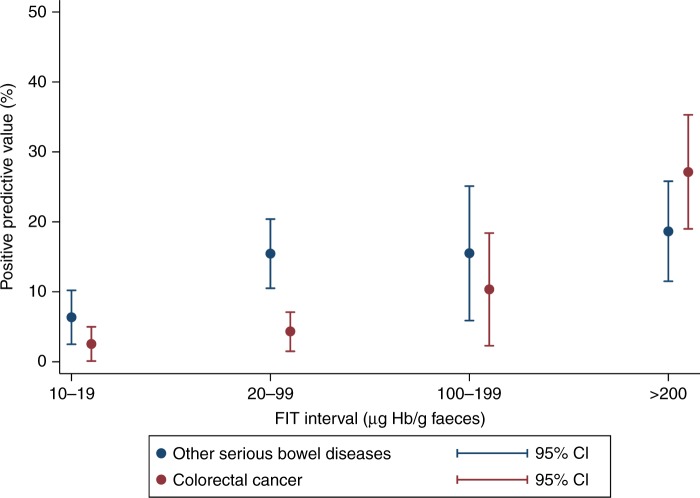
Positive predictive value for colorectal cancer and other serious bowel disease (inflammatory bowel disease and high-risk adenomas) stratified for faecal haemoglobin concentrations

The PPV for detecting CRC increased with increasing faecal haemoglobin concentration, whereas the PPV for SBD remained fairly constant for concentrations ≥20 µg Hb/g faeces (Fig. 2). The PPV for CRC with a FIT value of 10–19 µg Hb/g faeces was 2.5% (95%CI: 0.1;5.0), whereas this increased to 27.1% (95%CI: 19.0;35.3) for individuals with a FIT value of >200 µg Hb/g faeces. For SBD, the PPV was 6.4% (95%CI: 2.5;10.2) for a FIT value of 10–19 µg Hb/g faeces vs. 18.6% (95%CI: 11.5;25.8) for a FIT value of ≥200 µg Hb/g faeces.

## Discussion

### Main findings

This study is the first to investigate the clinical use of the FIT on individuals presenting with non-alarm symptoms of CRC in general practice. When the GP used the FIT, ∼16% of tests were positive; among these, 9.4% of patients were diagnosed with CRC and 13.5% with other serious bowel disease. For both CRC and SBD, the PPVs increased with age. However, females were more often diagnosed with SBD, whereas CRC was more frequent in males. There was no lower faecal haemoglobin concentration at which CRC or SBD did not occur.

Of the CRCs diagnosed after a positive FIT, 67% were diagnosed at stage I & II and 20% in stage IV. ∼40% of CRCs were located in the proximal colon. Less than three cases of CRC were found after 3 months of follow-up among individuals with negative FIT; this corresponds to a false negative rate of <0.1% for CRC.

The most frequently reported symptoms for requesting a FIT were change in bowel habits and abdominal pain. One-fifth of individuals with unexplained anaemia had a positive FIT; among these, 12% had CRC.

### Strengths and limitations

A major strength of this study was that the FIT was used in daily clinical practice. For approx. a decade, Danish GPs have been able to refer individuals with alarm symptoms of CRC to an urgent colonoscopy. The GPs in this study were instructed only to use the FIT on individuals with non-alarm symptoms of CRC. By doing this, we ensured that the GPs had a clearly defined diagnostic approach for each patient. By letting the GPs use their clinical judgement to decide on which patients to request the FIT, we believe that it is reasonable to assume that the results realistically reflect the use of the FIT on patients with non-alarm symptoms. We of course cannot be sure that this is actually the case, however, it is strongly supported by the fact that the population’s overall pre-test risk of CRC was 1.5%, which is below alarm symptoms, but higher than the baseline risk of CRC.^51^ The study was not designed as a study of diagnostic performance. However, this have been investigated in both screening and in general practice for individuals already referred to colonoscopy.^27,42^ We believe that it is reasonable to assume that the performance of the FIT in individuals with non-alarm symptoms will be somewhere in between these populations. Furthermore, the primary aim of this study was not to test the performance of FIT, but to assess whether the test would be of value in the diagnostic work up of individuals with non-alarm symptoms of CRC. For this purpose we believe that the design of the study was adequate.

Another strength was that the study was conducted at large scale and included ∼853 GPs who were all given the opportunity to use the FIT. However, fewer FITs were requested during the study period than expected. Most likely, this was due to an adaption period after implementation of the FIT and that some GPs (20%) did not start using the test during the study period. Nevertheless, we believe that the overestimation of FIT use primarily reflects the difference between register-based estimations and clinical reality.

The 3 months of follow-up ensured that we included all the CRCs and SBDs found in immediate relation to the FIT request, but it may also have underestimated both the PPVs and the false negative rate since some diagnoses may have occurred beyond the 3 months. However, the majority of symptomatic CRCs are seen in general practice in the months preceding diagnosis.^51^ Thus, it may be assumed that the majority of CRCs will have emerged within the follow-up time of this study. An additional source for underestimating the PPVs, was that 23% of individuals with a positive FIT did not have a diagnostic investigation performed within the first 3 months after performance of the FIT. Possible reasons for this may be that the GP, for relevant reasons, decided not to refer the patient to diagnostic investigation despite the positive FIT or that the GP missed or did not react to the test result after the analyses. Depending on the number of patients who were mistakenly not referred to diagnostic investigation, this might have underestimated the PPVs for CRC and SBD after a positive FIT. However, by using all individuals with a positive FIT for calculating the PPVs, we ensured an “intention-to-treat” analysis with known direction of a potential bias.

We did not find any emergency presentations of CRC during the study period. In contrast, studies from the UK indicate that >20% of annual CRC cases are diagnosed after emergency presentations.^52^ Our data did not hold information on the way the patient was admitted to the hospital. Therefore, we defined an emergency presentation as a CRC diagnosis, which was not preceded by a colonoscopy, sigmoidoscopy or CT-colonography, since the CRC most likely would have been diagnosed during a surgical procedure after emergency admittance to the hospital. This definition may have been too strict to identify all emergency presentations, but because our population had a low pre-test risk of CRC, we believe the number of emergency presentations would be small.

The results of this study are generalisable to similar settings as the Danish health care system, and can be used in the future planning of the diagnostic workup of patients with symptoms of CRC.

### Comparison with existing literature

A number of studies have assessed the use of FIT on symptomatic individuals, and the evidence for using the test in primary care is increasing.^22–30^ However, previous studies have mainly investigated the FIT use in a population already referred to colonoscopy from primary care. In contrast, our study explores using the FIT in a population for whom the GP does not find indication for referral to urgent colonoscopy. Therefore, we must assume the pre-test risk of CRC to be lower in our population and thus, specifically report on the use of FIT in individuals with low-risk symptoms on CRC. In 2015, an updated version of the NICE guideline’s referral for suspected cancer recommended testing for occult blood in faeces on individuals with low-risk symptoms on CRC.^6^ This update was widely criticised for using the older gFOBT in the recommendation. Thus, in 2017, the guideline was supplemented with a diagnostic guidance (DG30) suggesting using the FIT.^32,53^ However, the guidelines were conducted without any evidence of using FIT in individuals with low-risk symptoms of CRC. Therefore, we believe that the present results are the first to indicate that the decision to recommend faecal immunochemical testing on individuals with low-risk symptoms of CRC may have been right.

In addition to recommending the FIT as a diagnostic test for detecting CRC, the DG30 guidance, together with a range of other studies, have suggested using the FIT as a rule-out test.^23,26,27,53^ Though the FIT is generally believed to have a good performance in detecting CRC, our results suggest that false negative tests will occur even when using a low cut-off value. Therefore, choosing the diagnostic use of the FIT is a balance between preventing unnecessary investigations and not missing any diagnoses. No test will definitively rule out CRC and using the FIT as a rule-out test will inevitably result in missed CRC diagnoses. In our study, <15% of FIT negative individuals were referred for diagnostic investigation suggesting that GPs managed the FIT use well and used their clinical judgement and safety netting on each individual. We therefore suggest that the FIT should optimally be used as a rule-in test in individuals with non-alarm symptoms of CRC.

A recent study by Cubiella et al. have developed a prediction model to detect CRC in symptomatic patients by combining information on faecal haemoglobin concentration, age and gender (FAST score).^54^ In our study we found that the PPV for CRC and increased with age and faecal haemoglobin concentration, and were higher for males. Thus, our results support the findings of Cubiella et al. and underlines that each of these factors should be taken into account when interpreting a FIT result.

### Clinical use of the results

In total, 67% of CRCs were diagnosed in stage I & II and 20% in stage IV. These figures indicate that using the FIT on individuals with non-alarm symptoms of CRC may give a more favourable stage distribution of the CRCs compared to the current diagnostic pathway for symptomatic patients in general practice.^55^ However, this assumption is limited by the statistical precision in our study and more research is needed to make conclusions on this matter. Furthermore, we found that ∼40% of detected CRCs were located in the proximal colon; of these, 76% were diagnosed in stage I & II (results not shown). In general, proximal CRC is associated with poorer prognosis than distal CRC.^56^ Thus, this suggests that the FIT may be an important aid in diagnosing proximal CRC in early stages.

Symptoms and signs recommended for using the FIT were carefully selected from knowledge and literature on the presentation of CRC. We decided to recommend using the FIT on individuals with unexplained anaemia and change in bowel habits although these are normally considered alarm symptoms of CRC. However, the clinical reality for the GP is not black and white, and any symptom and sign can take different form of severity. Furthermore, anaemia is an often missed sign of CRC.^57,58^ It was a strict prerequisite for using the FIT that the GP did not find that the presented symptoms and signs met the criteria for urgent referral in the CPP for CRC. We found that unexplained anaemia was the indication with the highest positivity rate and PPV for CRC. Thus, this indicates that individuals with unexplained anaemia should at least have a FIT performed if the GP does not consider the individuals as eligible for urgent referral. From the present results, we cannot conclude whether a negative FIT will rule out CRC in individuals with unexplained anaemia, but since the population in the study in general is believed to have a low pre-test risk of CRC, the clinical value of a negative FIT is debatable. This was also the reason why we chose to use the FIT as a rule-in test.

We do not know to what extent the rates of colonoscopies were affected during the study period. However, we plan to investigate this in another study. It may be assumed that the rate would increase, but during the one year study period, 834 diagnostic investigations were performed. In comparison, more than 3000 colonoscopies and CT-colonography were performed during the initial 9 months of the Danish screening programme for CRC in the Central Denmark Region alone.^42^ Furthermore, the extra diagnostic investigations may be recovered in reduced expenses for treatment due to early detection of the CRC.^59^

## Conclusion

This study is the first to investigate the use of a safe, low-cost FIT in patients presenting with non-alarm symptoms of CRC in general practice. Our results suggest that the FIT may be used as a rule-in test in this group of patients to detect both CRC and SBD in primary care, and that the stage distribution of detected CRC by this method may be more favourable. However, awareness of false negative test results is important when using the FIT in this population, and further studies are needed to assess the exact performance of the FIT in this population.

Nevertheless, we consider the findings of importance in a realistic diagnostic work-up of patients with non-alarm symptoms of CRC and it reveals a possible diagnostic supplement for a group of patients that are notoriously difficult to handle in primary care.

## Availability of data and material

The datasets supporting the conclusions of this article are stored in a secured research database and may be available upon presentation of formal approval.
